# Decreased Brain pH and Pathophysiology in Schizophrenia

**DOI:** 10.3390/ijms22168358

**Published:** 2021-08-04

**Authors:** Hae-Jeong Park, Inyeong Choi, Kang-Hyun Leem

**Affiliations:** 1 Department of Pharmacology, School of Medicine, Kyung Hee University, Seoul 02447, Korea; 2 Department of Physiology, Emory University School of Medicine, Atlanta, GA 30322, USA; inyeong.choi@emory.edu; 3 Department of Herbology, College of Korean Medicine, Semyung University, Jecheon 27136, Korea

**Keywords:** schizophrenia, brain pH, lactate, mitochondria dysfunction, dopamine, glutamate, pH-regulating proteins

## Abstract

Postmortem studies reveal that the brain pH in schizophrenia patients is lower than normal. The exact cause of this low pH is unclear, but increased lactate levels due to abnormal energy metabolism appear to be involved. Schizophrenia patients display distinct changes in mitochondria number, morphology, and function, and such changes promote anaerobic glycolysis, elevating lactate levels. pH can affect neuronal activity as H^+^ binds to numerous proteins in the nervous system and alters the structure and function of the bound proteins. There is growing evidence of pH change associated with cognition, emotion, and psychotic behaviors. Brain has delicate pH regulatory mechanisms to maintain normal pH in neurons/glia and extracellular fluid, and a change in these mechanisms can affect, or be affected by, neuronal activities associated with schizophrenia. In this review, we discuss the current understanding of the cause and effect of decreased brain pH in schizophrenia based on postmortem human brains, animal models, and cellular studies. The topic includes the factors causing decreased brain pH in schizophrenia, mitochondria dysfunction leading to altered energy metabolism, and pH effects on the pathophysiology of schizophrenia. We also review the acid/base transporters regulating pH in the nervous system and discuss the potential contribution of the major transporters, sodium hydrogen exchangers (NHEs), and sodium-coupled bicarbonate transporters (NCBTs), to schizophrenia.

## 1. Introduction

Many neurotransmitters, including dopamine, serotonin, glutamate, and γ-aminobutyric acid (GABA), are involved in the pathophysiology of schizophrenia. In particular, dopamine plays an important role. Positive symptoms of schizophrenia (delusion, hallucination, and paranoia) result from the hyperfunction in the mesolimbic dopamine pathway, characterized by increased dopamine release and increased dopamine D2 receptor activation in the limbic regions [1,2]. Negative symptoms (amotivation, anhedonia, avolition, asociality, and flat affect) and cognitive symptoms of schizophrenia are caused by the hypofunction in the mesocortical dopamine pathway, characterized by decreased D1 receptor activation in the prefrontal cortex (PFC) [1,2]. Thus, antipsychotic drugs, which typically inhibit D2 receptors, control the positive symptoms but are less effective in alleviating the negative or cognitive symptoms [3], leading to incomplete remission of schizophrenia.

The pH in brains of schizophrenia patients is lower than normal [4,5,6,7]. The exact cause of this pH decrease is unclear, but altered energy metabolism due to mitochondria dysfunction appears to play a role [6,8,9,10,11]. The meta-analysis of human postmortem and magnetic resonance spectroscopy (MRS) studies provide evidence for altered energy metabolism, increased lactate, and decreased pH in schizophrenia brain [6]. Furthermore, the expressions of energy-metabolism-related genes are altered in the brains of schizophrenia patients [8]. pH is an important biological factor that can alter brain function and viability [12,13,14]. Numerous studies show that abnormalities in pH regulation are responsible for the pathophysiological changes of the brain [15,16]. A variety of CNS proteins such as neurotransmitter receptors, ion channels, and synaptic transmission machinery proteins are sensitive to pH, and a pH change can affect membrane excitability, action potential properties, and signaling cascades [12,16,17]. A low pH has both inhibitory and stimulatory effects on ion channels/receptors [16,18]. For example, a low pH blocks the voltage-gated Ca^2+^ channels and decreases Ca^2+^ accumulation in the presynaptic neurons [16], resulting in a suppression of neurotransmitter release. On the other hand, a low pH increases the amplitude and time course of GABA receptors [19] and activates acid-sensing ion channels ASICs to conduct Na^+^ and, in lower proportion, Ca^2+^ [18]. More importantly, a low pH also inhibits the N-methyl-d-aspartate (NMDA) glutamate receptors by reducing the open probability of the pore in the receptors and decreases membrane excitability [16]. This inhibition protects neurons from NMDA-mediated excitotoxicity [13]. Furthermore, mice with genetic deletion of some NCBTs such as NBCn1, NCBE, and NDCBE develop less excitable membranes, higher resting potential, or decreased glutamate release [20,21,22], and they are less susceptible to repetitive discharges in response to pentylenetetrazol, pilocarpine or NMDA [20,22]. Taken together, it is conceivable that the lowered brain pH in schizophrenia patients will influence synaptic activities involved in the pathophysiology of schizophrenia.

In this review, we discuss the current understanding of the phenomena of decreased brain pH in schizophrenia and its pathophysiological implications. The discussion is focused on mitochondria dysfunction serving as the prime cause of decreased pH, evidence from human samples and animal models of schizophrenia, and pH implication for schizophrenia pathophysiology based mainly on dopamine and glutamate hypotheses of schizophrenia. We also explain the pH-regulating acid base transporters in the brain and their potential roles in schizophrenia, particularly focusing on NHEs and NCBTs that have been extensively studied in CNS diseases. This review provides an overview of the current knowledge regarding abnormal brain pH in schizophrenia patients and can be a conceptual basis for future research on pH regulation in schizophrenia.

## 2. Decreased Brain pH in Patients with Schizophrenia

Neuroimaging techniques have been useful for assessing changes in energy metabolism [23,24]. For example, changes in glucose and oxygen consumption can be measured using oxygen or glucose PET and indirectly using fMRI. As for the brains of schizophrenia patients, changed energy metabolism has been assessed using MRS, which detects the presence and concentration of various metabolites [4,5,6,7,8,10]. In particular, phosphorus-31 MRS is a useful imaging technique to characterize the metabolic composition and allow quantitative assessment of molar concentrations of metabolites. This technique measures an altered ratio of ATP to phosphocreatine and other metabolites, as well as pH [4,5,6,7,8,10]. Postmortem and MRS imaging studies revealed that the pH in the brains of schizophrenia patients is on average 0.2 pH unit lower than controls [4,5,6,7]. This decrease is due to high levels of lactate [4,5,6,7,8,10]. The lactate levels in the PFC, cerebellum, and striatum of schizophrenia patients are 1.2–1.5-fold higher than controls [5,10,25]. Hagihara et al. [26] performed a meta-analysis on the postmortem brain pH of schizophrenia and found significantly decreased pH values in the schizophrenia samples. The authors also analyzed brain pH and lactate levels in mouse models of schizophrenia such as mice with gene knockout (KO) of schnurri-2, calcineurin, or neurogranin, and found a decrease in brain pH and an increase in lactate levels in the brains of these animals. Other researchers also found similar changes in different mouse models of schizophrenia, such as glutathione-deficient mice, NMDA receptor subunit 1 GluN1/Grin1 KO mice, and disrupted in schizophrenia1 (DISC1) KO mice [27,28]. Together, these reports demonstrate decreased brain pH and elevated lactate levels being the primary features of schizophrenia.

In contrast, other researchers argue that the decreased brain pH and increased lactate levels are not directly related to schizophrenia but instead could be secondary effects induced by antipsychotic drugs or agonal changes occurring just before death [5,29,30,31]. Halim et al. [5] examined pH and lactate levels in the cerebellum of schizophrenia patients and confirmed an inverse correlation between the two parameters, but found no correlation between lactate levels and age, postmortem interval, agonal state, or manner of death. The authors also observed decreased pH and elevated lactate levels in the frontal cortex of rats after chronic administration with clozapine or haploperidol [5]. Glavina et al. [32] reported increased blood lactate levels in patients with psychotic disorders who received haloperidol and olanzapine for 6 months, compared to the baseline lactate levels before treatment. The increase was also correlated with extrapyramidal symptoms (EPS) such as Parkinsonism and dystonia, which are side effects caused by antipsychotic drugs. Elmorsy et al. [33] found that both typical (chlorpromazine or haloperidol) and atypical antipsychotic drugs (risperidone, olanzapine, or quetiapine) increased blood lactate levels in patients with psychiatric disorders during the 3-month treatment period. In particular, chlorpromazine and haloperidol increased lactate levels within the first ten days of treatment and caused higher incidence of EPSs. The occurrence and severity of EPSs were positively correlated with lactate levels.

Conclusively, it is evident that schizophrenia patients have decreased brain pH and increased lactate levels. These changes are recapitulated in a variety of mouse models of schizophrenia and validated as the primary features of schizophrenia. On the other hand, the changes can also be induced by chronic administration of antipsychotic drugs. Thus, the changes occur primarily due to the pathologic conditions of schizophrenia and secondarily as a result of antipsychotic drug treatments. Regardless, mitochondria dysfunction plays a key role in these changes, as discussed in the following section.

## 3. Mitochondria Dysfunction in Schizophrenia

The mitochondria are responsible for the production of high-energy ATP from glucose via the oxidative phosphorylation system (OXPHOS), also known as mitochondrial respiration. Because the brain is one of the highest energy-demanding organs in the body and heavily depends on glycolysis and OXPHOS to produce ATP, mitochondria dysfunction in the brain can lead to neurodevelopmental and neuropsychiatric problems [9,34]. Numerous studies have reported mitochondria dysfunction in schizophrenia [8,9,35,36,37,38,39,40,41]. This abnormality may account for the increased lactate levels and subsequent pH decrease in the brains of schizophrenia patients [6,9,10,11]. In this section, we will focus on the morphological and functional abnormalities in mitochondria related to energy metabolism in schizophrenia.

Schizophrenia patients have lower numbers of mitochondria in the striatum [38]. The number and volume of mitochondria are also low in the PFC and caudate nucleus, particularly in oligodendrocytes [41]. The morphological changes in mitochondria are also observed in peripheral cells. For example, schizophrenia patients have a lower density of mitochondria in activated lymphocytes than controls [40]. The patients also have fewer and swelled mitochondria with fragmentation of the cristae in mononuclear cells [36].

Mitochondria are also functionally altered in schizophrenia. For example, cytochrome C oxidase in the mitochondrial electron transport chain (ETC) displays reduced activity in the frontal cortex, caudate nucleus, and temporal cortex of schizophrenia patients [35]. Cytochrome C oxidase and ATP synthase are also decreased in the hippocampus [8]. Both mRNA and protein levels of the 24-kDa and 51-kDa protein subunits of complex I in the ETC are decreased in the PFC, while no change is observed in the 75-kDa subunit expression [37]. The activity of complex IV is significantly reduced in the frontal cortex and temporal cortex of schizophrenia patients [39]. Moreover, there is an abnormal energy metabolism in schizophrenia [42,43,44,45,46,47]. There are increased phosphodiester and decreased phosphomonoester levels in the frontal cortex of schizophrenia patients [43,44,45,46,47], indicating lipid breakdown. In addition, the levels of phosphocreatine, a rapid mobilizable reserve of high-energy phosphates, are increased in the temporal lobe [42].

The abovementioned changes in mitochondria morphology and function indicate that the production and maintenance of high-energy ATP via OXPHOS are not normal in schizophrenia. Impaired OXPHOS causes anaerobic glycolysis and converts pyruvate to lactate, which utilizes NADH to NAD and produces H^+^ [9,48,49]. Furthermore, antipsychotic drugs can alter mRNA and protein levels of mitochondrial proteins in OXPHOS [50]. The drugs not only inhibit the ETC but also downregulate ATP synthase subunits, resulting in inability to maintain cellular ATP levels. Thus, the drugs exert additional effects on the already dysfunctional mitochondria, accounting for increased lactate levels and decreased pH observed after chronic antipsychotic drug treatments discussed in the above section. In addition, abnormal oxidative reactions in mitochondria constantly produce reactive oxygen species (ROS) that induce apoptosis [51,52], inflammation [53,54], and mutations in mitochondrial DNA [55], all of which contribute to the pathogenesis of schizophrenia [11,56,57,58,59]. The production of ROS worsens abnormal energy metabolism and can exacerbate high lactate levels [11].

It is worthwhile to discuss how close the relationship between energy metabolism and brain function is. Korf and Gramsbergen [60] addressed the temporal relationship between energy usage and neuronal activity and found that energy usage is slow to significantly contribute to the metabolic responses associated with fast neurotransmission. Stojanov et al. [61] have argued that neural activities in milliseconds do not depend on energy recruitment but use energy resources that are already available as potential energy. The authors further proposed that energy metabolism in the brain is used primarily to maintain and restore potential energy, rather than being involved directly in higher brain functions such as memory retrieval, speaking, and consciousness. While this is an interesting idea, at present there is no report on changed potential energy (such as imbalance of Na^+^, K^+^, and other ion components) in the brains of schizophrenia patients. Attwell and Laughlin [62] have discussed that energy usage depends strongly on action potential rate and a large fraction of the total energy used by the brain is expended on signaling. With respect to decreased pH mediated by lactate production, a local pH difference cannot be excluded in the brains of schizophrenia patients. The brains express different types of pH-regulating proteins in cell and tissue-specific manners, and such variations in expression can have steady and prolonged impacts on neuronal activity depending on different brain regions. This is consistent with the fact that the pH-regulating proteins play an important role in connecting energy metabolism and neuronal activity.

## 4. Effects of Low Brain pH on Neuronal Activity in Schizophrenia

### 4.1. Dopaminergic and Glutamatergic Systems

Decreased brain pH can alter a variety of CNS functions as numerous proteins are sensitive to pH [12,16,17]. With respect to schizophrenia, decreased pH can cause changes in the two features: an increase in synaptic dopamine levels and a decrease in NMDA receptor activity.

Studies of lactic acid application to rat striatum show increased synaptic dopamine levels by lactic acid [63,64]. Lactic acid perfusion into rat striatum increases extracellular concentrations of dopamine and catabolites [64]. Lactic acid also impairs dopamine uptake in rat striatal slices and synaptosomes [63]. Impairment of dopamine uptake is immediate upon lactic acid incubation and can be maintained after the washout of lactic acid. The maintained impairment is related to peroxidative damage, but additional factors appear to be involved. Increased dopamine undergoes auto-oxidation and is converted to dopaquinone, which irreversibly binds to cysteinyl residues in the dopamine transporters and inhibits dopamine uptake [65,66,67]. In the lactic acid experiments described above, the authors used lactic acid at pH 5.3, markedly lower than the pH change in schizophrenia. It is unclear whether similar changes in dopamine levels are induced in brains of schizophrenia patients. However, a small but persistent pH decrease may affect steady-state dopamine release or uptake at synapses. The outcome is in good agreement with the hyperfunction of dopaminergic neurons in the mesolimbic system responsible for positive symptoms of schizophrenia [1,2].

The most important impact of decreased brain pH on schizophrenia would be mediated by altering NMDA receptor function. NMDA receptors play important roles in the pathophysiology of schizophrenia [68,69]. The NMDA receptor antagonists phencyclidine (PCP) and ketamine induce schizophrenic-like conditions in healthy subjects [70,71] and worsen the symptoms in schizophrenia patients [72]. Furthermore, PCP and MK-801 are widely used to induce schizophrenia in rodents [73,74,75,76]. Paradoxically, the systemic administration of PCP or MK-801 to rodents increases glutamate release in the PFC [73,74,75,76]. Schizophrenia patients also show elevated glutamate levels in the anterior cingulate cortex and striatum [77,78,79,80]. The increase in glutamate levels is seemingly odd; however, additional studies reveal that the antagonists inhibit the NMDA receptors located outside the PFC, possibly in GABAergic neurons that tonically inhibit glutamatergic inputs to the PFC [70,71,74]. Inhibition of the NMDA receptors by PCP or MK-801 decreases GABAergic inhibition of glutamatergic inputs, and as a result glutamate release is increased. NMDA receptors are sensitive to brain pH as their activities are suppressed by extracellular acidification [12]. The half maximum inhibition of the receptors is pH 7.3 [81], which is close to physiological pH in the brain. The 0.2 pH unit decrease in schizophrenia is thus expected to cause a substantial inhibition of NMDA receptor activity. The impact of this inhibition on schizophrenia is comparable to the NMDA receptor antagonists and should depend upon the tissue specific expression of the receptor subunits, particularly pH-sensitive GluN2B subunit [82], in the neural network associated with schizophrenia.

Conclusively, a persistently low brain pH in schizophrenia will cause a small but constant imbalance of dopaminergic and glutamatergic transmission, and as a result symptoms of schizophrenia may be emerged. In addition, increased neurotransmitter release from presynaptic vesicles increases H^+^ accumulation at synapses as the vesicles release H^+^ and neurotransmitters together, producing rapid acidification of the synaptic cleft [18]. Additionally, intensive synaptic activity may increase the energy demand from astrocytes [18], which increases lactic acid production. Lactic acid is transported to the extracellular space by monocarboxylate transporters (MCTs) and lowers extracellular pH [18].

### 4.2. Noradrenergic System

Studies show elevated norepinephrine levels in the cerebrospinal fluid (CSF) and blood of schizophrenia patients [83,84]. There is compelling evidence that the positive and negative symptoms of schizophrenia are correlated with hyperactivity and hypoactivity of the noradrenergic system, as reviewed by Yamamoto et al. [85]. Low pH affects chemosensitive noradrenergic neurons of the locus coeruleus (LC) [86], which respond to changes in blood pH or partial pressure of carbon dioxide (PCO_2_). Acidification can depolarize LC neurons and increase their firing rates [86,87]. The LC noradrenergic system, which provides the widespread distribution and branching of the noradrenergic neurons throughout the whole brain, releases not only norepinephrine but also dopamine in the cerebral cortex (including PFC) and hippocampus [88,89,90,91,92]. Thus, in schizophrenia patients, the low brain pH may alter dopamine release from LC noradrenergic neurons in these regions. Low brain pH may also affect serotonergic neurons of the raphe nucleus [93], which are chemosensitive neurons responding to changes in blood pH or PCO_2_.

## 5. Possible Involvement of pH-Regulating Proteins in Schizophrenia

As in other cells, steady-state pH in neurons is maintained by the balance between acid loading and acid extrusion rates [15,94]. Table 1 shows a list of pH-regulating proteins in the brain. In neurons, acid loading is typically mediated by the chloride-bicarbonate exchanger AE3/SLC4A3 while acid extrusion is mediated by the sodium-hydrogen exchangers NHE1/SLC9A1, NHE3/SLC9A3, and NHE5/SLC9A5, and the sodium-coupled bicarbonate transporters (NCBTs) NBCn1/SLC4A7, NDCBE/SLC4A8, and NCBE/NBCn2/SLC4A10 [15]. In addition to these major pH-regulating proteins, other proteins can also affect intracellular pH depending upon types of neurons. Here, we will discuss the potential roles of pH-regulating proteins in schizophrenia, focusing on NHEs and NCBTs that have been extensively studied in CNS diseases.

### 5.1. NHEs

NHEs mediate an electroneutral exchange of one Na^+^ for one H^+^ across the plasma membrane [16,97]. There are nine isoforms; NHE1–5 are predominantly localized to plasma membranes [16,98,99] and NHE6–9 are in subcellular organelles [15,100]. NHE3 and NHE5 are additionally found in endosomal pools [16,98,99]. The NHEs in the brain are primarily NHE1, NHE3, and NHE5.

#### 5.1.1. NHE1 and NHE3

Dopamine activates NHE1 via D2-like receptors in multiple cell lines [101,102]. Nerve et al. [102] found that dopamine and the D2 receptor agonist quinpirole induced extracellular acidification in C6 glioma cells and L fibroblasts expressing D2 receptor. The NHE inhibitor amiloride abolished the extracellular acidification without affecting D2 receptor activity. Chio et al. [101] observed a similar result in CHO cells expressing D4 receptor. Extracellular acidification was induced by dopamine and quinpirole and abolished by the dopamine receptor antagonists clozapine and spiperone. In contrast, dopamine inhibits NHE3 in the kidney tissues and cells [103,104,105,106]. In the renal proximal tubule epithelia, where NHE3 was predominantly expressed, dopamine regulated Na^+^ transport through the inhibition of NHE3 [103], Na/K-ATPase [107], and NBCe1 [108]. Similarly, dopamine inhibits NHE3 and Na/K-ATPase in the opossum proximal tubule cell line OK cells [105]. The inhibition of NHE3 by dopamine is mediated by cAMP-dependent protein kinase A (PKA) [105,106], but both PKA and PKC signaling cascades appear to be involved [104].

Dopamine receptors are G-protein-coupled receptors, and the G protein activation affects adenylyl cyclase. D1-like receptors stimulate adenylyl cyclase and increase cAMP levels, whereas D2-like receptors inhibit adenylyl cyclase and inhibit cAMP production. This means that the dopamine-mediated NHE3 inhibition occurs through the activation of D1-like receptors. In contrast, the stimulation of NHE1 by D2-like receptors in C6 and L cells is not associated with adenylyl cyclase [102]. Recently, Richards et al. [109] reported that NHE1 can be activated by cGMP and inhibited by cAMP in cardiomyocytes. Thus, dopamine inhibits NHE3 by cAMP via D1-like receptor activation but stimulates NHE1 by cGMP via D2-like receptor activation.

It is unclear whether alterations in NHE1 and NHE3 activities are observed in schizophrenia. Given that dysfunction of dopamine transmission is a major hypothesis for schizophrenia, it is conceivable that a pH change due to abnormal activities of NHE1 and NHE3 occurs in the brains of schizophrenia patients and affects the pathophysiology of schizophrenia, as well as the action of antipsychotic drugs.

NHE1 is expressed in both neurons [110,111] and astrocytes [112], and broadly found in many different brain regions [15,113]. NHE3 is found in cerebellar Purkinje cells [15,113] and the brain stem/medulla oblongata, where it is highly expressed in chemosensitive neurons [114,115]. These expression profiles imply that NHE1 likely plays a role in pH regulation of the schizophrenia-affecting regions such as PFC and mesolimbic/mesocortical pathways. The Na^+^ transport by NHE1 is known to alter intracellular environments and influence the expression and activity of other membrane transporter proteins [16]. Loss of NHE1 increases excitability of the CNS [116] as it causes higher Na^+^ channel expression and activity in neurons [117,118]. It is postulated that abnormalities in dopamine release or uptake in schizophrenia may alter NHE1 activity by activating D2-like receptors and change intracellular acidification in neurons. This change may then affect membrane excitability of neurons.

#### 5.1.2. NHE5

NHE5 is highly expressed at synapses in most of the brain regions and responsible for synaptic pH regulation during an action potential. NMDA receptor recruits NHE5 from endosomes to plasma membranes [110], promoting an acidification in the synaptic cleft. Recruited NHE5 inhibits dendritic spine growth that is stimulated by NMDA receptor activation. Interestingly, NHE5 KO mice display enhanced learning and memory [119]. In the hippocampus of these mice, the levels of synaptic proteins such as synaptophysin and post-synaptic density protein 95 (PSD95) were higher and the brain-derived neurotrophic factor (BDNF)/tropomycin receptor kinase B (TrkB) signaling pathway was activated [119]. BDNF, which is a member of the neurotrophin family, binds to TrkB receptor and activates neuronal maturation, synapse formation, and synaptic plasticity. Thus, NHE5 affects dendritic spine growth and neuronal synaptic formation by modulating BDNF/TrkB signaling.

One might expect that the plasma membrane expression of NHE5 should be low in schizophrenia with hypofunction of NMDA receptors and BDNF levels should be elevated. There are clinical and experimental reports that ketamine or MK-801 elevates BDNF and TrkB levels [120,121,122]. However, the elevation was transient and did not last. These results are inconsistent with low levels of BDNF and TrkB receptor in schizophrenia patients. The postmortem brains of schizophrenia patients show decreased levels of BDNF and TrkB receptor mRNA and proteins, particularly in the PFC and hippocampus [123,124,125]. BDNF levels are also low in the blood from schizophrenia patients [126,127,128,129]. The effect of NHE5 on BDNF/TrkB signaling might be complicated in schizophrenia.

#### 5.1.3. NHE6 and NHE9

NHE6 is primarily found in early recycling endosomes and regulates organellar pH and Na^+^ content [130]. NHE6 extrudes luminal H^+^ that was pumped into endosomes by the V-ATPase, and thus is responsible for maintaining a steadily acidic pH in early endosomes. NHE6 is widely found in human brain, with the highest expression in the hippocampus [131]. NHE6 mutations are one of the most recurrent mutations in patients with X-chromosome linked intellectual disability [132]. NHE6/SLC9A6 mutations lead to neurological phenotypes associated with syndromic autism, broadly characterized into overlapping clinical categories: Christianson syndrome [133], Angelman-like syndrome [134], corticobasal degeneration with tau deposition [135], and epilepsy. Moreover, a missense mutation in NHE6 has been identified as one of rare mutations in only one schizophrenic individual or patients with a common ancestry [136]. NHE6 KO mice develop accelerated acidification of endosomes and reduced levels of axonal and dendritic arborization [137], hallmarks for intellectual and developmental disabilities. Defects in arborization are due to decreased TrkB levels and phospho-Trk induction after BDNF, indicating that endosomal signaling through BDNF/TrkB is perturbed.

NHE9 is found in late recycling endosomes and found in both neurons and astrocytes [130]. NHE9/SLC9A9 gene variants in humans are closely associated with attention deficit hyperactivity disorder (ADHD), autism spectrum disorders, and addiction [138,139,140,141,142]. Consistent with these genetic data, NHE9 KO mice develop decreased ultrasonic vocalization, decreased preference for social novelty, and increased self-grooming [143], all of which are characteristics of autistic behaviors. Similar to NHE6, NHE9 regulates plasma membrane distribution and recycling of receptors and transporters [131,144,145]. Typically, NHE9 mutations associated with autism accelerate endosomal acidification and degrade glutamate aspartate transporter (GLAST) [145]. This results in decreased plasma membrane expression of GLAST and elevated synaptic glutamate level.

It is presently unclear whether abnormalities in NHE6 or NHE9 are associated with schizophrenia, but circumstantial evidence for such an association exists as described above. The perturbation of BDNF/TrkB signaling in NHE6 KO mice is consistent with low levels of BDNF and TrkB receptor in schizophrenia patients [123,124,125]. Increased synaptic glutamate levels due to decreased GLAST membrane expression in NHE9 mutations is consistent with increased synaptic glutamate levels [73,74,75,76,77,78,79,80]. Antipsychotic drugs reduce the increased synaptic levels of glutamate [74,146].

### 5.2. NCBTs

NCBTs move Na^+^ and HCO_3_^−^ into cells and extrude intracellular H^+^ as HCO_3_^−^ interacts with intracellular H^+^ [15,17,147,148]. In the brain, NBCn1, NCBE (NBCn2), and NDCBE are highly expressed in neurons, whereas NBCe1 and NBCe2 are primarily in astrocytes [15,17]. NBCn1 is predominantly localized to postsynaptic membranes [17,149], where it regulates postsynaptic and synaptic cleft pH. NDCBE is localized to presynaptic vesicles and regulates glutamate release in a pH-dependent manner [22,150]. NCBE expression is colocalized to pre- and post-synaptic markers of GABAergic synapses in hippocampal neurons [14]. Knockouts of these neuronal NCBTs protect mice from seizures incidence and mortality in response to pentylenetetrazol, pilocarpine, or NMDA [14,20,22].

Among neuronal NCBTs, NBCn1 and NDCBE affect glutamate neurotransmission [20,21,22,149] and thus are of particular interest with respect to their potential involvement in schizophrenia. NBCn1 interacts with the NMDA receptor subunit GluN1 and constitutes a protein complex in the postsynaptic density [20,151]. NBCn1 KO mice develop downregulation of GluN1 and PSD95 in the hippocampal neurons, resulting in decreased NMDA excitotoxicity and seizure [20]. Mice with reduced GluN1 levels display increased locomotor activity and stereotypy and deficits in social and sexual interactions [152]. These behavioral abnormalities are similar to those in PCP and MK801-treated mice and can be improved by haloperidol or clozapine treatment. Conditional knockout of GluN1 in the nucleus accumbens attenuates apomorphine-induced D1 receptor trafficking and diminishes auditory-evoked startle and social interactions [153], which are impaired in multiple psychiatric disorders including schizophrenia. Thus, given the molecular and functional association of GluN1 with NBCn1, there is a strong possibility that NBCn1 plays a role in schizophrenia. As for NDCBE, electrophysiological analysis and FM-imaging show a decrease in spontaneous and stimulated release of glutamatergic synaptic vesicles in NDCBE KO neurons [22]. Taken together, given that the impairment of glutamatergic activity particularly in the mesolimbic pathway affects schizophrenic behaviors [68,69,154,155,156,157,158], it is postulating that NBCn1 and NDCBE play a role in schizophrenia by altering glutamate release or NMDA receptor activity.

## 6. Model for pH Involvement in Schizophrenia

We discussed in the above sections the current understanding of decreased brain pH in schizophrenia and its pathophysiological implications. The decreased pH is due to mitochondria dysfunction as impaired OXPHOS leads to anaerobic glycolysis and converts pyruvate to lactate, producing H^+^. A persistently low brain pH is expected to cause a prolonged imbalance of dopaminergic and glutamatergic transmission. Furthermore, intensive synaptic activity accelerates extracellular acidification in the synaptic cleft. The pH-regulating transporters, particularly NHEs and NCBTs, will respond to such pH changes to compensate acidification, which alters dopamine or glutamate release or uptake. Conversely, abnormal release or uptake of these neurotransmitters may alter NHEs or NCBTs. Overall, the potential roles of pH-regulating proteins in schizophrenia are not trivial, and their abnormal activities may affect the pathophysiology of schizophrenia.

**Figure 1:**
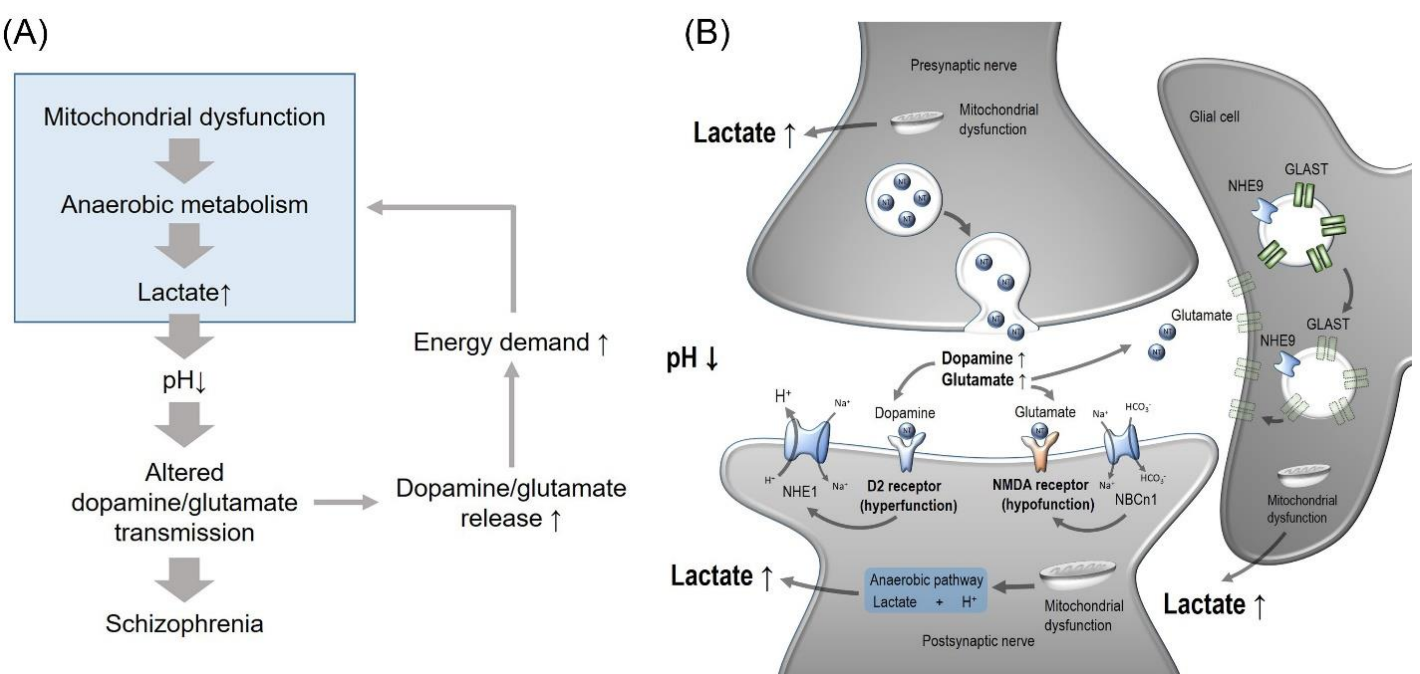
Model for the effect of decreased brain pH on schizophrenia. (**A**) Overview of the model. Mitochondrial dysfunction in schizophrenia causes anaerobic metabolism that elevates lactate levels, resulting in lactic acidosis. Acidosis alters dopamine and glutamate neurotransmission, causing symptoms of schizophrenia. Enhanced release of dopamine and glutamate may conversely increase an energy demand in neurons and astrocytes, promoting elevated production of lactic acid, which aggravates acidosis. (**B**) Synaptic pH regulation in schizophrenia. Enhanced dopamine release and hyperactivated D2 receptors stimulate NHE1, which compensates intracellular acidification while inducing extracellular acidification. At glutamatergic synapses, NBCn1 in postsynaptic neurons decreases the NMDA receptor activity by preventing the interaction with the receptors. NHE9 in astrocytes increases the plasma membrane expression of glutamate transporters to increase glutamate uptake.

Based on the current information on brain pH function, a model for pH involvement in schizophrenia can be made as shown in Figure 1. The main concept of the model is that the decreased brain pH alters dopamine and glutamate transmission (Figure 1A). It increases dopamine release and inhibits dopamine uptake, promoting dopaminergic transmission responsible for the positive symptoms of schizophrenia. Increased transmission requires an energy demand from neurons and astrocytes, which additionally increases anaerobic metabolism. Brain has delicate pH regulatory systems to maintain normal extracellular and CSF pH, and acid extruders will operate to compensate the acidic milieu in schizophrenia. A model for the synaptic pH regulation by NHEs and NCBTs under such conditions is illustrated in Figure 1B. Enhanced dopamine release and hyperactivated D2 receptors stimulate NHE1, which compensates intracellular acidification while inducing extracellular acidification. At glutamatergic synapses, NBCn1 in postsynaptic neurons decreases the NMDA receptor activity by preventing the interaction with the receptors. Furthermore, NHE9 in astrocytes increases the plasma membrane expression of glutamate transporters to increase glutamate uptake. In this sense, NHE9 acts to relieve schizophrenia symptoms. It is possible that NHE6 could be defective and causes low levels of BDNF and TrkB receptor. In summary, the model proposes that the decreased brain pH in schizophrenia triggers dopamine transmission and hypofunction of NMDA receptors and stimulates acid-extruding NHEs and NCBTs. These changes will influence synaptic activities involved in the pathophysiology of schizophrenia.

## 7. Conclusions

The average brain pH in schizophrenia patients is lower than normal. pH can affect numerous proteins essential for neuronal activity, and it is conceivable that the decreased brain pH in schizophrenia has substantial impacts on vulnerability of cognitive symptoms in schizophrenia. The decreased brain pH is expected to trigger dopamine transmission by increasing steady-state dopamine release or interfering with uptake at synapses, particularly in the mesolimbic system responsible for positive symptoms of schizophrenia. The decreased brain pH is also expected to induce hypofunction of NMDA receptors, which decreases GABAergic inhibition of glutamatergic inputs to the PFC and results in increased glutamate release. This phenomenon is comparable to the increased glutamate release by the NMDA receptor antagonists. Brain will respond to decreased pH and stimulate the acid extruders NHEs and NCBTs for the maintenance of pH homeostasis. The impact of such changes on schizophrenia pathophysiology will depend upon the tissue-specific expression of individual members of NHEs and NCBTs in the neural network associated with schizophrenia. 

## Figures and Tables

**Table 1:** Major pH-regulating proteins in the CNS.

Protein	Type	Function	Reference
Na^+^/H^+^ exchanger	NHE1–9	Exchange extracellular Na^+^ for intracellular H^+^	[95]
Na^+^-coupled HCO_3_^−^ transporter	NBCe1, e2, NBCn1, n2/NCBE	Cotransport Na^+^ and HCO_3_^−^ into cells	[15,95]
	NDCBE	Move Na^+^ and HCO_3_^−^ into cells in exchange for intracellular Cl^−^	[17]
Cl^−^/HCO_3_^−^ exchanger	AE1–3	Exchange intracellular HCO_3_^−^ for extracellular Cl^−^	[95]
Vacuolar type H-ATPase	V-ATPase	Move H^+^ from cytoplasm into vesicles or extracellular space by using ATP	[95]
Monocarboxylate transporter	MCT1, 2, 4	Cotransport H^+^ and monocarboxylate anion (such as lactate, pyruvate, acetoacetate, and/or β-hydroxybutyrate)	[95,96]
Carbonic anhydrase	CA1–15	Catalyze the inter conversion of CO_2_ and H_2_O to HCO_3_^−^ and H^+^	[95]

## Data Availability

Not applicable.
